# Phytochemical and Biological Studies of *Agave attenuata*

**DOI:** 10.3390/ijms13056440

**Published:** 2012-05-24

**Authors:** Komal Rizwan, Muhammad Zubair, Nasir Rasool, Muhammad Riaz, Muhammad Zia-Ul-Haq, Vincenzo de Feo

**Affiliations:** 1 Department of Chemistry, Government College University, Faisalabad 38000, Pakistan; E-Mails: komal.rizwan45@yahoo.com (K.R.); zubairmkn@yahoo.com (M.Z.); nasirhej@yahoo.co.uk (N.R.); riaz_453@yahoo.com (M.R.); 2 Research Institute of Pharmaceutical Sciences, Department of Pharmacognosy, University of Karachi, Karachi 75270, Pakistan; E-Mail: ahirzia@gmail.com; 3 Department of Pharmaceutical and Biomedical Sciences, University of Salerno, Via Ponte don Melillo, I-84084 Fisciano (Salerno), Italy

**Keywords:** *Agave attenuata*, GC-MS, peroxidation, volatile fraction, flavonoids, phenolics

## Abstract

The present study was conducted to examine various biological activities of a methanol extract of *Agave attenuata* leaves. GC-MS analysis of the *n*-hexane fraction from the extract revealed the presence of 31 compounds, with mono-2-ethylhexyl phthalate (11.37%), 1,2-benzenedicarboxylic acid (6.33%), *n*-docosane (6.30%) and eicosane (6.02%) as the major components. The leaves contained appreciable levels of total phenolic contents (10.541–39.35 GAE, mg/100 g) and total flavonoid contents (43.35–304.8 CE, mg/100 g). The extract and some of its fractions showed moderate antimicrobial effects. Leaves extract and fractions also exhibited a good antioxidant potential when measured by DPPH radical scavenging activity and inhibition of lipid peroxidation assays. The hemolytic effect of the plant was found to be in a range of 1.01%–2.64%. From the present study it is concluded that this plant could be used as a source of natural antioxidants and functional food nutraceutical applications.

## 1. Introduction

Plants possess an almost limitless ability to biosynthesize phytochemicals, which serve as a source for natural antioxidants and as plant defense mechanisms against predation by microorganisms, herbivores and insects [1,2]. Gupta [3] asserted that in comparison to synthetic drugs, antimicrobials and antioxidants of plant origin are not associated with many side effects and has an enormous therapeutic potential to heal many infections and diseases.

Many species of the genus *Agave* constitute an important source of steroidal sapogenins, mainly hecogenin [4]. In the pharmaceutical industry, these natural compounds are used for the semisynthesis of medicinal steroids, as corticosteroids, sexual hormones and steroid diuretics [5]. The most investigated species of the genus is *A. americana* L. that has been reported for its antibacterial and anti-inflammatory properties [6]. Some other species possess anticancer properties [7]. Antimicotic, antiviral and antituberculosis properties have also been ascribed to this genus. The antimicrobial activity of some species against pathogens has been previously reported [8,9]. Some species of this genus are used in Chinese Traditional Medicine in treatment of scabies, tumors, dysentery, and as insecticides [10–12].

*Agave attenuata* Salm-Dyck is native to central Mexico and tropical America. The plant is commonly grown as a garden plant. In fact, the plant has no teeth or terminal spines; this property makes it an ideal plant for areas adjacent to footpaths. It is a hardy survivor of drought, tolerating heat well and moderate salt exposure. Previous literature reports showed the presence in the plant of steroidal saponins [13–15]. The plant may provide a substitute for niclosamide and be used safely for snail control by rural communities [16] and has been proposed as a contact poison for *Bulinus africanus* [17].

Despite its multipurpose uses, very little data exists on its chemical constituents; therefore the current study reports the chemical composition of *n*-hexane fraction of *A. attenuta* leaves and the *in vitro* antimicrobial, antioxidant, hemolytic activities of the methanol extract and fractions thereof. This study will provide base-line data for further detailed investigations of various biological activities of this plant and of its use as a functional food.

## 2. Results and Discussion

### 2.1. GC-MS Analysis of *n*-Hexane Fraction

The GC-MS analysis of *n*-hexane fraction from methanol extract enabled the identification of 31 components, representing 90.32% of the total fraction. This volatile fraction consisted of a mixture of different classes of compounds. The major constituents were found to be mono-2-ethylhexyl phthalate (11.37%), 1,2-benzene dicarboxylic acid (6.33%), *n*-docosane (6.30%), and eicosane (6.02%) (Table 1). Gutierrez *et al*. [18] reported the chemical composition of a lipophilic extract of *Agave sisalana* fiber. Most of *n*-alkanes and fatty acids identified in *A. sisalana* fiber were also identified in volatile fraction of *A. attenuata* leaves.

### 2.2. Antioxidant Potential of *A. attenuata* Leaves

The amounts of Total Phenol Content (TPC) and Total Flavonoid Content (TFC) extracted with different solvents from leaves of *A. attenuata* are reported in Table 2. These values ranged from 10.54 to 39.35 gallic acid equivalents (GAE) (mg/100 g of dry plant matter) and from 304.8 to 43.35 catechin equivalents (CE) (mg/100g of dry plant matter), respectively. The ability of different solvents to extract TPC was found as follows: methanol (39.35%) > chloroform (19.3%) > ethylacetate (14.5%) > *n*-butanol (10.65%) > *n*-hexane (10.54%).

The ability of solvents on TFC values was found in the following order: 100% methanol (304.8%) > ethylacetate (197.2%) > chloroform (79.8%) > *n*-butanol (71.5%) > *n*-hexane (43.35%). The DPPH radical scavenging activity of extract and fractions (0.1 mg/mL) ranged from 61.41 to 73.97%. Methanol extract showed the highest scavenging activity (73.97%), followed by ethyl acetate (73.36%), *n*-butanol (65.21%), chloroform (64.94%) and *n*-hexane (61.41%) fractions, respectively. The percent inhibition of linolei c acid oxidation ranged from 50.12% to 70.35%, whereas BHT provided inhibition at the level of 84.7%. The methanol extract exhibited the highest inhibition of linoleic acid oxidation (70.35%) followed by ethylacetate (67.78%), *n*-butanol (66.23%), chloroform (60.25%) and *n*-hexane (50.12%) fractions, respectively. When the results of DPPH scavenging activity and the percent inhibition of linoleic acid oxidation were compared with standard BHT, all the samples showed significantly (*p* < 0.05) minor activity. The reducing potential of the tested extract and fractions was observed at concentrations of 1 mg/mL. The absorbance recorded for the tested samples in this assay resulted in the range of 0.219–0.664 nm [19]. The maximum absorbance (0.664 nm) was recorded for chloroform fraction, while the minimum was for *n*-hexane (0.20 nm) one. The reducing power of different extracts and fractions decreased in the following order: chloroform > 100% methanol > *n-*butanol > ethylacetate > *n*-hexane. When these results were compared with standard ascorbic acid (0.8 nm), all the samples showed a minor reducing power (Table 2). Results indicated that among all the extract and fractions; the methanolic extract of plant leaves exhibited the highest amount of TPC and TFC, and correspondingly the highest antioxidant activity as measured by DPPH radical scavenging and inhibition of linoleic acid oxidation; whereas, *n*-hexane fraction demonstrated the least antioxidant activity probably because of its low polarity [20].

### 2.3. *In Vitro* Hemolytic Activity

Hemolytic activity was studied because, even if the plant possesses potent antioxidant and antimicrobial activities, its use in medicine will be impossible in the presence of these hemolytic effects linked to the presence of saponins. The total hemolysis (100%) was obtained using 20 μL of Triton X-100 (0.1%). Chloroform fraction showed the highest hemolytic effect (2.64%) [21], followed by *n*-hexane (2.09%), methanol extract (1.46%), ethylacetate (1.40%) and *n*-butanol (1.01%) fractions. The hemolytic effect of *n*-butanol fraction was less than other fractions (Table 3). However, data obtained are in a safe range and the plant extracts may be safe for human use.

### 2.4. Antimicrobial Activity

The plant exhibited considerable antimicrobial activity against all the strains tested as reported in Table 4. The results from the disc diffusion method, followed by measurement of minimum inhibitory concentration (MIC), indicated that the methanol extract showed strong inhibitory activity against *A. flavus* and *A. alternata*, with the highest inhibition zones (27.5 and 20.75 mm) and the lowest MIC values (18.4 and 69.4 mg/mL). Methanol extract showed no activity against *P. multocida* and *A. niger.* Chloroform fraction showed strong inhibitory action against *R. solani* and *A. flavus* with high inhibition zones (26.7 and 19 mm) and reduced MIC values (20.4 and 25.1 mg/mL). A weak activity was exhibited against *P. multocida*, and *S. aureus*. Ethylacetate fraction showed good inhibitory activity only against *E. coli*, with an inhibition zone of 18.75 mm and the lowest MIC value (94.2 mg/mL). Ethylacetate fraction was inactive to inhibit the growth of *A. flavus*, *A. alternata* and *R. solani. n*-Butanol fraction showed a strong activity against *E. coli* with highest inhibition zone (26.8 mm) and the lowest MIC value (15.2 mg/mL) if compared to the standard drug rifampicin whose inhibition zone was of 21.5 mm (MIC 62.1 mg/mL). Little activity was exhibited against *B. subtilis*, with the small inhibition zone (13 mm) and the highest MIC value (170 mg/mL). *n*-hexane fraction showed strong inhibitory action against *P. multocida*, with the inhibition zone (25.75 mm) and the lowest MIC value (27.4 mg/mL); this fraction resulted inactive against *B. subtilis*.

Sanchez *et al*. [8] reported antifungal activity of *A. asperrima* and *A. striata* flowers extracts against *A. flavus* for both agave methanol extracts with a better antifungal effect. These results are in agreement with our analysis where methanol extract exhibited a strong inhibitory action against *A. flavus* and good inhibitory effects against all tested fungal strains. *A. flavus* causes many diseases both in humans and animals as aspergillosis of the lungs and sometimes causing corneal, otomycotic, and nasoorbital infections.

## 3. Experimental

### 3.1. Plant Material

The leaves of the plant *A. attenuata* were collected on April 2010 from the Botanical Garden, University of Agriculture, Faisalabad, Pakistan. The plant specimens were further identified by Mansoor Hameed, Department of Botany University of Agriculture Faisalabad, Pakistan where a voucher specimen has been deposited.

### 3.2. Preparation of Extract and Organic Fractions

Four kilograms of fresh leaves were washed with distilled water to remove dust and other extraneous matter. The shade dried leaves were powdered (80 mesh). Five hundred grams of powdered leaves were extracted at room temperature for 5 days with 3 L of methanol. The extract was filtered through Whatman No. 1 filter paper and then concentrated at 45 °C, using a rotary vacuum evaporator. The methanol extract was then stored at −4 °C. The residue (85 g) was dissolved in distilled water and extracted successively with *n*-hexane (30 g residue), chloroform (15 g) ethylacetate (20 g), and *n*-butanol (15 g).

### 3.3. Gas Chromatography/Mass Spectrometry Analysis

The GC-MS analysis of the *n*-hexane fraction was performed using GC 6850 Network gas chromatographic system equipped with 7683 B series auto injector and 5973 inert mass selective detector (Agilent Technologies USA). Compounds were separated on an HP-5 MS capillary column having 5% phenyl polysiloxane as stationary phase, column length 30.0 m, internal diameter 0.25 mm and film thickness 0.25 μm. The temperature of injector was 300 °C and 1.0 μL of sample was injected in the split mode with split ratio 30:1. Helium was used as carrier gas, with a flow rate of 1.5 mL/min. The temperature program was: initial temperature 150 °C and held for 1 min, then ramping at rate of 10 °C/min up to 290 °C and finally hold at this temperature for 5 min. The temperature of MSD transfer line was 300 °C. For mass spectra determination MSD was operated in electron ionization (EI) mode, with the ionization energy of 70 eV, while the mass range scanned was 3–500 *m*/*z*. The temperature of ion source was 230 °C and that of MS quadropole 150 °C. The identification of components was based on comparison of their mass spectra with those of NIST mass spectral library [22–23].

### 3.4. Antimicrobial Assay

#### 3.4.1. Test Microorganisms

*Aspergillus niger* ATCC 10595, *Aspergillus flavus* ATCC 32612, *Alternaria alternata* ATCC 20084, *Rhizoctonia solani* locally isolated, were used as the fungal tested organisms and *Pasturella multocida* locally isolated, *Escherichia coli* ATCC 25922, *Bacillus subtilis* JS 2004, *Staphylococcus aureus* API Staph tac 6736153 were used as the bacterial tested organisms. The pure bacterial and fungal strains were obtained from the Department of Veterinary Microbiology, University of Agriculture, Faisalabad, Pakistan. The bacterial strains were cultured overnight at 37 °C in nutrient agar (Oxoid, Hampshire, UK) while fungal strains were cultured overnight at 28 °C using potato dextrose agar (Oxoid).

#### 3.4.2. Disc Diffusion Method

Antimicrobial activity of the methanol extract and of its fractions was determined by using the disc diffusion method [24]. All samples (dry residue) were dissolved in 10% sterile dimethyl sulfoxide. The discs (6 mm diameter) were impregnated with 20 mg/mL extract/fractions (100 μL/disc) placed aseptically on the inoculated agar. Discs injected with 100 μL of respective solvents served as a negative controls, rifampcin (100 μL/disc) (Oxoid) and fluconazole (100 μL/disc) (Oxoid) were used as positive reference for bacteria and fungi, respectively. The petri dishes were incubated at 37 ± 0.1 °C for 20–24 h and 28 ± 0.3 °C for 40–48 h for bacteria and fungi, respectively. At the end of period, the inhibition zones formed on the media were measured. The positive antimicrobial activity was read based on growth inhibition zone.

#### 3.4.3. Resazurin Microtitre-Plate Assay

The minimum inhibitory concentration (MIC) of the plant extract/fractions was evaluated by a modified resazurin microtitre-plate assay reported by Sarker and co-workers [25] with some modifications. Briefly, a volume of 100 μL of each extract and fractions solution in 10% dimethyl sulfoxide (DMSO, *v*/*v*) was transferred into the first row of the 96 well plates. To all other wells, 50 μL of nutrient broth and Muller Hinton broth for bacteria and fungi respectively were added. Two-fold serial dilutions were performed using a multichannel pipette such that each well had 50 μL of the test material in serially descending concentrations. To each well, 10 μL of resazurin indicator solution (prepared by dissolving 270 mg resazurin tablet in 40 mL of sterile distilled water) were added. Finally, 10 μL of bacterial/fungal suspension were added to each well. Each plate was wrapped loosely with aluminum foil. Each plate had a set of controls: a column with broad spectrum antibiotics as positive control, a column with all solutions with the exception of the test samples, a column with all solutions with the exception of the bacterial/fungal solution adding 10 μL of broths instead and a column with respective solvents as a negative control. The plates were prepared in triplicate, and incubated at 37 ± 0.1 °C for 20–24 h and 28 ± 0.3 °C for 40–48 h for bacteria and fungi, respectively The absorbance was measured at 620 nm by micro quant for fungus and at 500 nm for bacteria. The color change was then assessed visually. The growth was indicated by color changes from purple to pink or colorless. The lowest concentration at which color change appeared was taken as the MIC value.

### 3.5. *In Vitro* Hemolytic Activity

Hemolytic activity of the plant was checked by the reported method of Powell and co-workers [26]. Three milliliter of freshly obtained heparinized human blood was gently mixed, poured into a sterile 15 mL polystyrene screw-cap tube and centrifuged for 5 min, at 850 *g*. The supernatant was poured off and the viscous pellet washed three additional times with 5 mL of chilled (4 °C) sterile isotonic phosphate-buffered saline (PBS) solution, adjusted to pH 7.4. The washed cells were suspended in a final volume of 20 mL chilled, sterile PBS and the cells counted on a haemacytometer. The blood cell suspension was maintained on wet ice and diluted with sterile PBS to 7.068 × 10^8^ cells mL^−1^ for each assay. Aliquots of 20 μL of plant extract/fractions were aseptically placed into 2.0 mL microfuge tubes. For each assay, 0.1% Triton X-100 was used as the positive, 100% lytic control and PBS as the negative, 0% lytic control. Aliquots of 180 μL diluted blood cell suspension were aseptically placed into each 2-mL tube and gently mixed three times with a wide mouth pipette tip. Tubes were incubated for 35 min at 37 °C with agitation (80 revolutions per minute). Immediately following incubation, the tubes were placed on ice for 5 min, then centrifuged for 5 min at 1310 *g*. Aliquots of 100 μL of supernatant were carefully collected, placed into a sterile 1.5 mL microfuge tube, and diluted with 900 μL chilled, sterile PBS. All tubes were maintained on wet ice after dilution. Then 200 μL were placed into 96 well plates, and three replicates was taken in well plate which contain one positive and one negative. Absorbance at 576 nm was then measured on a microquant. The experiment was done in triplicate. Percent hemolysis was calculated by following formula:

%hemolysis=Abs (sample absorbance)/Abs (control absorbance)×100

### 3.6. Evaluation of Antioxidant Activity

#### 3.6.1. Determination of Total Phenolic Contents (TPC)

Amount of TPC were assessed using Folin–Ciocalteu reagent procedure [27]. Briefly, 50 mg of dry mass of crude extract/fraction was mixed with 0.5 mL of Folin-Ciocalteu reagent and 7.5 mL deionized water. The mixture was kept at room temperature for 10 min, and then 1.5 mL of 20% sodium carbonate (*w*/*v*) was added. The mixture was heated in a water bath at 40 °C for 20 min and then cooled in an ice bath; finally absorbance at 755 nm was measured (Hitachi U-2001 spectrophotometer). Amounts of TP were calculated using a calibration curve for gallic acid (10–100 ppm) (*R*^2^ = 0.9986). The results were expressed as gallic acid equivalents (GAE) of dry plant matter.

#### 3.6.2. Determination of Total Flavonoid Contents (TFC)

The Total Flavonoid Content (TFC) in plant extract and fractions was determined following the procedure as described by Dewanto and co-workers [28]. Plant extract/fractions of each material (1 mL containing 0.1 g/mL) was placed in a 10 mL volumetric flask, then added distilled water 5 mL and 0.3 mL of 5% NaNO_2_ was added to each volumetric flask initially; after 5 min., 0.6 mL of 10% AlCl_3_ was added. After another 5 min, 2 mL of 1 M NaOH was added and volume made up with distilled water. Then solution was mixed. At 510 nm absorbance of the reaction mixture was taken using a spectrophotometer. TFC were evaluated as catechin equivalents (g/100 g of dry plant matter).

#### 3.6.3. DPPH Radical Scavenging Assay

The 2,2-diphenyl-1-picrylhydrazyl radical (DPPH) assay was carried out spectrophotometrically as described by Bozin and co-workers [29]. The antioxidant activity of the methanol extract and various fractions (0.1 mg/mL) were mixed with 1 mL of 90 μM DPPH solution and made up with 95% methanol, to a final volume of 4 mL. After 1 h incubation period at room temperature, the absorbance was recorded at 515 nm. Inhibition of free radical by DPPH in percent (%) was calculated in the following way:

$$\text{Inhibition }(\%)=100\times ({\text{Abs}}_{\text{blank}}-{\text{Abs}}_{\text{sample}}/{\text{Abs}}_{\text{blank}})$$

where Abs_blank_ is the absorbance of the control (containing all reagents except the test samples), and Abs_sample_ is the absorbance of the test samples.

#### 3.6.4. Antioxidant Activity in Linoleic Acid System

The antioxidant activity of *A. attenuata* leaves methanol extract and its fractions was also determined in terms of measurement of percent inhibition of peroxidation in linoleic acid system following a method reported by Iqbal and Bhanger [30]. Extract/fractions (5 mg) were added to a solution mixture of linoleic acid (0.13 mL), 99.8% ethanol (10 mL) and 10 mL of 0.2 M sodium phosphate buffer (PH 7.0). Total mixture was diluted to 25 mL with distilled water. The solution was incubated at 40 °C and the degree of oxidation was measured following thiocyanate method [28] with 10 mL of ethanol (75%), 0.2 mL of an aqueous solution of ammonium thiocyanate (30%), 0.2 mL of sample solution and 0.2 mL of ferrous chloride (FeCl_2_) solution (20 mM in 3.5% HCl) being added sequentially. After 3 min of stirring, the absorption values of mixtures measured at 500 nm were taken as peroxide contents. A control was performed with linoleic acid but without extracts. Synthetic antioxidants butylated hydroxytoluene (BHT) and ascorbic acid (200 ppm) were used as positive control. The maximum peroxidation level observed at 360 h (15 days) in the sample that contained no antioxidant component was used as a test point. The percent inhibition of linoleic acid peroxidation was calculated as follows:

$$\text{Percent inhibition of linoleic acid peroxidation}=100-\frac{\text{Abs}.\;\text{increase of sample at }360\;\text{h}}{\text{Abs}.\;\text{increase of control at }360\;\text{h}}\times 100$$

#### 3.6.5. Determination of Reducing Power

The reducing power of the extract/fractions was determined according to the procedure described by Yen and co-workers [31] with little modification. Equivalent volume of leaves crude extracts/fractions containing 1.0 mg of dry matter was mixed with sodium phosphate buffer (5.0 mL, 0.2 M, pH 6.6) and potassium ferricyanide (5.0 mL, 1.0%); the mixture was incubated at 50 °C for 20 min. Then 5 mL of 10% trichloroacetic acid was added and centrifuged at 980 *g* for 10 min at 5 °C in a refrigerated centrifuge (Centrifuge H-200NR, Kokusan, Japan). The upper layer of the solution (5.0 mL) was diluted with 5.0 mL of distilled water and ferric chloride (1.0 mL, 0.1%) and absorbance noted at 700 nm (Hitachi U-2001 Spectrophotometer).

### 3.7. Statistical Analysis

Each sample was analyzed individually in triplicate for its data were reported as mean (*n* = 3 × 3 × 1) ± standard deviation (*n* = 3 × 3 × 1). Data were analyzed by analysis of variance (ANOVA) using Minitab 2000 Version 13.2 statistical software (Minitab Inc., Pennysalvania, USA).

## 4. Conclusions

Data obtained in the present study confirmed the considerable biological activities possessed by *A. attenuata*. The presence of biologically important phytochemicals in the plant extracts may contribute to their medicinal value and potential sources for useful drugs. The investigated plant may be processed for pharmaceutical and natural therapies for the treatment of ailments in humans. Further the antioxidant properties exhibited by this plant indicate its possible use as a functional ingredient for processing into health foods in the food industry.

## Figures and Tables

**Table 1:** Chemical constituents of *A. attenuata* leaves *n*-hexane fraction.

Retention Time	Compounds	Area%
3.338	Benzothiazole	0.94
4.186	Tetradecane	0.79
4.686	*n-*Undecane	2.12
5.005	*n*-Pentadecane	2.91
5.711	(E)-1-Methoxymethoxy-1-tetradecane-3-ol	0.88
5.915	*n*-Hexadecane	2.28
6.029	1,2-Benzenedicarboxylic acid	6.33
6.877	*n*-Heptadecane	2.60
7.856	*n*-Octadecane	2.51
7.958	Phytane	1.29
8.339	2-Undecanone	0.57
8.823	Nonadecane	2.42
9.090	Hexadecanoic acid	3.68
9.437	Palmitic acid	4.57
9.773	Icosane	2.43
10.695	Heneicosane	3.44
10.752	9-Octadecanoic acid	3.65
10.951	Octadecanoic acid	0.60
11.099	Linoleic acid	2.57
11.577	*n*-Docosane	6.30
12.430	Eicosane	6.02
13.255	Tetracosane	2.01
14.052	Pentacosane	4.30
14.541	Mono-2-ethylhexyl phthalate	11.37
14.820	*n*-Hexacosane	3.98
15.657	*n*-Octacosane	0.60
15.861	Tetracosanoic acid	0.76
17.398	Nonacosane	3.57
18.536	*n*-Triacontane	2.15
19.366	2,5-Cyclohexadiene-1,4-dione	0.85
19.879	Nonadecane	1.83
**Total**		**90.32%**

**Table 2:** Antioxidant potential of the methanol extract and its fractions from *A. attenuata* leaves.

Extract or Reference Compound	Total Phenolic Contents (GAE mg/100 g)	Total Flavonoid Contents (CE mg/100 g)	DPPH (% inhibition, 0.1 mg/mL)	Inhibition in Linoleic Acid System (%)	Reducing Power (Absorbance, nm 1 mg/mL)
Methanol	39.35 ± 0.69 ^a^	304.8 ± 5.02 ^a^	73.97 ± 1.49 ^b^	70.35 ± 1.34 ^b^	0.631 ± 0.016 ^b^
Chloroform	19.3 ± 0.91 ^b^	79.8 ± 3.91 ^c^	64.94 ± 0.85 ^c^	60.25 ± 1.02 ^d^	0.664 ± 0.012 ^b^
Ethylacetate	14.56 ± 1.11 ^c^	197.2 ± 4.96 ^b^	73.36 ± 0.94 ^b^	67.78 ± 1.64 ^b,c^	0.487 ± 0.015 ^b^
*n*-Butanol	10.65 ± 1.24 ^d^	71.5 ± 2.96 ^c^	65.21 ± 0.98 ^c^	66.23 ± 1.82 ^c^	0.625 ± 0.019 ^b^
*n*-Hexane	10.54 ± 0.40 ^d^	43.35 ± 2.99 ^d^	61.41 ± 1.07 ^d^	50.12 ± 1.12 ^e^	0.219 ± 0.013 ^c^
Ascorbic Acid	-	-	-	-	0.8 ± 0.021 ^a^
BHT	-	-	90.3 ± 2.04 ^a^	84.3 ± 2.33 ^a^	-

Data are expressed as the mean ± standard deviation; values having different letters differ significantly (*p* < 0.05).

**Table 3:** Hemolytic activity, as a percentage of hemolysis caused by *A. attenuate* leaves methanol extract, its fractions and standard.

Plant Extract Fraction	% of Hemolysis
100% Methanol	1.46 ± 0.14
Chloroform	2.64 ± 0.11
Ethylacetate	1.40 ± 0.04
*n*-Butanol	1.01 ± 0.04
*n*-Hexane	2.09 ± 0.08
Phosphate Buffer Saline (PBS)	0
Triton X-100	100 ± 0.61

**Table 4:** Antimicrobial activity of a methanol extract and fractions of *A. attenuata* leaves.

Tested Microorganism	Methanol Extract and Its Fractions (Diameter of Inhibition Zone, mm)					Standard Drugs	

	Methanol	Chloroform	Ethylacetate	*n*-Butanol	*n*-Hexane	Rifampcin	Fluconazole
*B. subtilis*	14.0 ± 1.41 ^b^	11.5 ± 1.11 ^c,d^	10.0 ± 1.58 ^d^	13.0 ± 0.70 ^b,c^	0	30.0 ± 1.41 ^a^	n.d
*P. multocida*	0	10.0 ± 0.70 ^d^	12.5 ± 1.118 ^d^	16.5 ± 1.65 ^c^	25.75 ± 2.58 ^b^	29.75 ± 0.43 ^a^	n.d
*S. aureus*	9.75 ± 0.43 ^c^	10.0 ± 0.70 ^c^	10.75 ± 0.78 ^c^	19.25 ± 0.43 ^b^	10.5 ± 0.5 ^c^	31.75 ± 2.04 ^a^	n.d
*E. coli*	17.0 ± 1.22 ^b,c^	14.2 ± 0.82 ^c^	18.75 ± 0.43 ^a^	26.8 ± 0.62 ^b^	14.25 ± 2.27 ^c^	21.5 ± 2.06 ^a^	n.d
*A. niger*	0	10.2 ± 1.47 ^b^	10.5 ± 1.11 ^b^	16.3 ± 1.65 ^a^	16.5 ± 1.68 ^b^	n.d	18.5 ± 1.11 ^a^
*A. flavus*	27.5 ± 2.5 ^b^	19 ± 0.707 ^c^	0	19.2 ± 0.82 ^c^	17.0 ± 1.00 ^c^	n.d	1.0 ± 1.00 ^a^
*A. alternata*	20.75 ±0.43 ^b^	14.5 ± 1.5 ^c^	0	19.5 ± 0.43 ^b^	12.25 ± 0.5 ^d^	n.d	25.5 ± 1.18 ^a^
*R. solani*	16.50 ± 1.65 ^d^	26.7 ± 2.38 ^b^	0	20.0 ± 0.707 ^c^	14.75 ± 2.94 ^d^	n.d	30.25 ± 0.43 ^a^

**Minimum Inhibitory Concentration (MIC) mg/mL.**							

*B. subtilis*	158 ± 1.15	205 ± 2.71	250 ± 2.10	170 ± 1.15	0	8.45 ± 0.25	n.d
*P. multocida*	0	250 ± 2.47	185 ± 1.25	115 ± 1.28	27.4 ± 0.64	9.5 ± 0.45	n.d
*S. aureus*	252 ± 1.45	250 ± 2.35	220 ± 2.40	89.3 ± 0.84	240 ± 2.45	5.32 ± 0.15	n.d
*E. coli*	110 ± 0.75	152 ± 0.79	94.2 ± 0.87	15.2 ± 1.15	140 ± 1.45	62.1 ± 0.45	n.d
*A. niger*	0	244 ± 2.24	240 ± 2.85	118 ± 1.12	115 ± 1.15	n.d	98.2 ± 0.55
*A. flavus*	18.4 ± 0.75	25.1 ± 0.55	0	89.5 ± 0.52	110 ± 1.52	n.d	6.4 ± 0.25
*A. alternata*	69.4 ± 0.25	142 ± 0.25^c^	0	85.4 ± 0.75	189 ± 0.95	n.d	27.4 ± 0.15
*R. solani*	115 ± 0.75	20.4 ± 0.75	0	80.2 ± 0.85	140 ± 0.75	n.d	7.8 ± 0.16

Data are expressed as the mean ± standard deviation; values having different letters differ significantly (*p* < 0.05) (n.d. = not detected).
